# Survival Impact of Primary Tumor Lymph Node Status and Circulating Tumor Cells in Patients with Colorectal Liver Metastases

**DOI:** 10.1245/s10434-017-5818-2

**Published:** 2017-03-03

**Authors:** Lars Thomas Seeberg, Cathrine Brunborg, Anne Waage, Harald Hugenschmidt, Anne Renolen, Ingunn Stav, Bjørn A. Bjørnbeth, Elin Borgen, Bjørn Naume, Kristoffer W. Brudvik, Gro Wiedswang

**Affiliations:** 1 Department of Gastrointestinal Surgery, Oslo University Hospital, Oslo, Norway; 2 Oslo Centre of Biostatistics and Epidemiology, Research Support Services, Oslo University Hospital, Oslo, Norway; 3 Department of Pathology, Oslo University Hospital, Oslo, Norway; 4 Department of Oncology, Oslo University Hospital, Oslo, Norway; 5 Institute of Clinical Medicine, Oslo University Hospital, Oslo, Norway; 6 Department of Gastrointestinal Surgery, Vestfold Hospital Trust, Tønsberg, Norway

## Abstract

**Objective:**

The aim of this study was to analyse the survival impact of primary tumor nodal status (N0/N+) in patients with resectable colorectal liver metastases (CLM), and to determine the value of circulating and disseminated tumor cells (CTCs/DTCs) in this setting.

**Methods:**

In this prospective study of patients undergoing resection of CLM from 2008 to 2011, peripheral blood was analyzed for CTCs using the CellSearch System^®^, and bone marrow was sampled for DTC analyses just prior to hepatic resection. The presence of one or more tumor cells was scored as CTC/DTC-positive. Following resection of the primary tumor, the lymph nodes (LNs) were examined by routine histopathological examination.

**Results:**

A total of 140 patients were included in this study; 38 patients (27.1%) were negative at the primary colorectal LN examination (N0). CTCs were detected in 12.1% of all patients; 5.3% of patients in the N0 group and 14.7% of patients in the LN-positive (N+) group (*p* = 0.156), with the LN-positive group (N+) consisting of both N1 and N2 patients. There was a significant difference in recurrence-free survival (RFS) when analysing the N0 group versus the N+ group (*p* = 0.007) and CTC-positive versus CTC-negative patients (*p* = 0.029). In multivariate analysis, CTC positivity was also significantly associated with impaired overall survival (OS) [*p* = 0.05], whereas DTC positivity was not associated with survival.

**Conclusion:**

In this cohort of resectable CLM patients, 27% had primary N0 colorectal cancer. Assessment of CTC in addition to nodal status may contribute to improved classification of patients into high- and low-risk groups, which has the potential to guide and improve treatment strategies.

**Electronic supplementary material:**

The online version of this article (doi:10.1245/s10434-017-5818-2) contains supplementary material, which is available to authorized users.

In surgery for colorectal cancer (CRC), primary tumors are resected with curative intent, if possible. However, more than 25% of CRC patients with localized disease at diagnosis will die as a result of cancer relapse,^1^^,^ 2 with liver being the most frequent metastatic site.^1^^–^ 3

Postoperative staging of the primary cancer is based on histological examination of the specimen, traditionally according to the tumor, node, metastases (TNM) staging system.^4^ Currently, the indication for adjuvant treatment is founded on this staging as colon cancer patients with lymph node (LN) involvement are offered chemotherapy postoperatively. Although the prognosis of patients with node-positive disease is inferior to those with node-negative disease, the outcome differs highly within the same TNM stage.^5^ In fact, between 25 and 40% of patients with primary node-negative CRC will develop liver metastases.^5^^–^ 9

Traditionally, the development of metastases has been understood as a sequential cancer progression where malignant transformation of the epithelial cells initially takes place in the intestinal mucosa, then infiltrates into the intestinal wall and therafter secondary LN involvement, and, subsequently , metastasize to distant organs. Accordingly, a high-risk patient for future relapse of a non-metastatic cancer will then be a patient with LN involvement.^10^^,^ 11 However, in agreement with recent understanding of cancer biology, cancer cell dissemination may occur at all stages of cancer development.^12^^,^ 13

The presence of circulating tumor cells (CTCs) is a significant prognostic factor in both non-metastatic^14^ and metastatic CRC.^15^^–^ 17 Disseminated tumor cells (DTCs) in the bone marrow (BM) have a prognostic impact in CRC patients, at least in long-term follow-up.^18^ The aim of the present study was to determine the relationship between primary tumor nodal status and CTC and DTC status in relation to survival in patients resected for colorectal liver metastases (CLM).

## Methods

### Patients

**Fig. 1:**
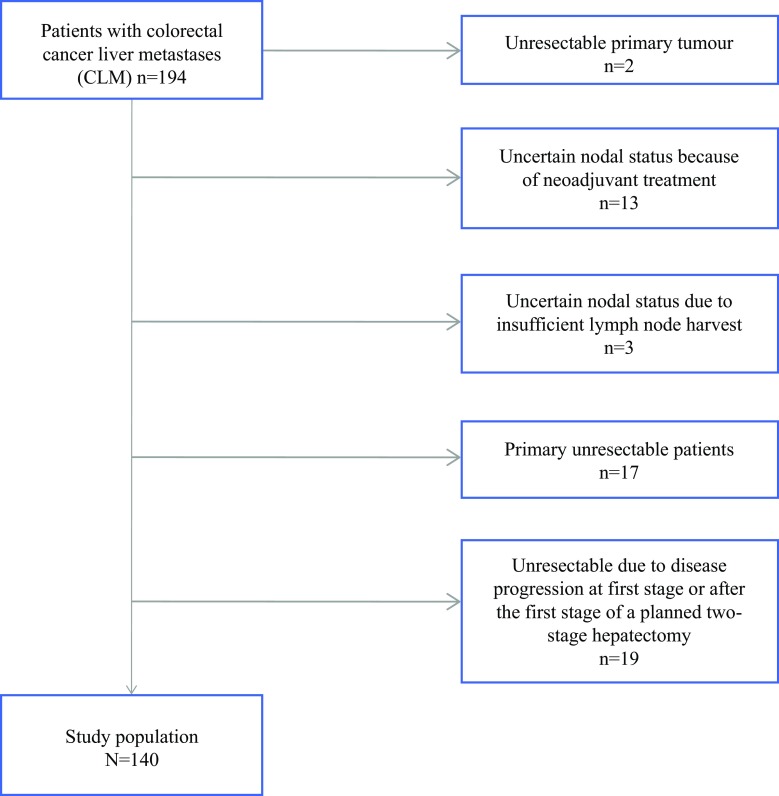
Study selection process for the cohort of 194 CLM patients, selecting the actual study population of 140 patients with resectable CLM. *CLM* colorectal liver metastases

Patients included in this study represent a subgroup from a prospective cohort study of 194 CLM patients referred to Oslo University Hospital for surgical treatment from May 2008 to December 2011. This cohort has been previously described in detail by Seeberg et al.^15^ In order to obtain a homogenous study population of CLM patients with resectable liver metastases and reliable LN status for the present analysis, two patients without resection of their primary tumor, and hence unknown nodal status, were excluded. Thirteen of the 60 node-negative patients had received neoadjuvant radiotherapy and/or chemotherapy before resection of the primary tumor, and were excluded because of uncertain nodal status. Another three patients had an inadequate number of lymph nodes resected (<8), according to the present Norwegian guidelines,^19^ and were therefore excluded. In addition, 17 patients were evaluated as primary unresectable for their liver metastases. In 19 patients, the liver metastases were non-resectable peroperatively or due to progression after the first-stage liver resection. After exclusion of all these patients, 140 patients remained and were eligible for the present analysis (Fig. 1). Clinical follow-up consisted of regular consultations every 4 months, with clinical and radiological assessments. Recurrence was determined as radiologically-proven relapse of the disease.

### Ethics/Study Approval

All patient characteristics were registered prospectively in a database using the Filemaker Pro Advanced documentation (Santa Clara, CA, USA). The study protocol was approved by the Regional Ethical Committee in Oslo, Norway, and the Oslo University Hospital Patient-Surveillance Service. The database was approved by the Data Protection Officer for Research, and permission for biobanking was obtained from the National Health Department.

### Circulating Tumor Cell (CTC) Detection

The US FDA-approved CellSearch System (Janssen Diagnostics LLC, Raritan, NJ, USA) was used for CTC detection. The samples were taken under general anesthesia, just prior to liver resection from peripheral blood. The method has been previously described in detail.^20^ Blood samples were collected into CellSave tubes, one tube per patient (7.5 ml), and the samples were kept at room temperature and processed at the Micrometastasis Laboratory, Oslo University Hospital, within 96 h of collection. CTC positivity was defined as one or more CTCs detected per sample of blood.^21^

### Bone Marrow Preparation and Disseminated Tumor Cell (DTC) Detection

The BM aspirations were performed at the same time as the CTC sampling, by bilateral aspiration from the crista iliaca anterior (5 ml BM from each site), and processed as previously described.^22^ As the CellSearch System is not recommended or validated for BM analyses, immunocytochemical detection was performed manually by density centrifugation using ficoll-hypaque to isolate the BM mononuclear cells, followed by the preparation of cytospins. The spins were immunostained using anticytokeratin antibodies AE1/AE3, and screened using the Ariol SL-50 automated screening system to identify epithelial cancer cells.^22^^,^ 50 The same number of slides were analyzed with an irrelevant control antibody to rule out false positives. All immunopositive candidate cells were evaluated by a pathologist (EB), and only cells with immune morphology satisfying the standard criteria for DTCs were scored as positive. DTC positivity was defined as one or more DTCs detected, and thus DTC detection is based on a different system than CTC detection with separate sensitivity and specificity.

### Pathological Examination

The specimens were analyzed at the local hospital, assessing tumor differentiation, resection margins, and T status. No assessment of KRAS status or microsatellite instability were performed. LNs exceeding 3 mm were divided into two or more parts parallel to their long axis, and all nodes were examined by routine microscopy, i.e. in 3–4 μm hematoxylin and eosin-stained sections. Patients were registered as being LN-positive (N+) or LN-negative (N0); the N+ group included both N1 (67 patients) and N2 (35 patients) [Table 1]. According to the Norwegian guidelines at the time,^23^ immunohistochemistry or polymerase chain reaction (PCR) analyses were not routinely performed.

**Table 1:** Characteristics of the CLM patient cohort [*n* = 140]

	N0	N+	*p*-Value
	[*n* = 38] (%)	[*n* = 102] (%)	
Age, years (±SD)	64.4 (11.0)	65.1 (9.2)	0.71^a^
Sex			0.81^b^
Males	21 (55.3)	54 (52.9)	
Females	17 (44.7)	48 (47.0)	
Primary			0.007^b^
Colon	32 (84.2)	61 (59.9)	
Rectum	6 (15.8)	41 (40.1)	
T status			0.001^c^
T2	11 (28.9)	3 (2.9)	
T3	25 (65.8)	82 (80.4)	
T4	1 (2.6)	15 (14.7)	
Nodal status^e^	38 (27.1)	102 (72.9)	
CEA (range)	3.5 (1–106)	6 (1–1381)	0.06^d^
Liver metastases			0.002^c^
Synchronous	15 (39.5)	70 (68.7)	
Metachronous	23 (60.5)	32 (31.3)	
Neoadjuvant^f^			0.004^c^
Yes	15 (39.5)	66 (66.7)	
No	23 (60.5)	33 (33.3)	
Adjuvant^g^			0.14^c^
Yes	21 (55.3)	68 (68.7)	
No	17 (44.7)	31 (31.3)	
CTCs			0.16^b^
Positive, ≥1 CTC	2 (5.3)	15 (14.7)	
Negative	36 (94.7)	87 (85.3)	
DTCs			0.73^b^
Positive, ≥1 DTC	4 (10.5)	8 (7.8)	
Negative	34 (89.5)	94 (92.2)	
Death			0.27^c^
Yes	7 (18.4)	28 (27.5)	
No	31 (81.6)	74 (72.5)	
Recurrence			0.008^c^
Yes	19 (50.0)	75 (73.5)	
No	19 (50.0)	27 (26.5)	

*CLM* colorectal liver metastases, *SD* standard deviation, *CEA* carcinoembryonic antigen, *CTCs* circulating tumor cells, *DTCs* disseminated tumor cells

^a^ Independent sample *t*-test

^b^ Fisher’s exact test

^c^ Pearson’s *Chi* square test

^d^ Mann–Whitney *U* test. The following variables have missing values: T status, *n* = 3; neoadjuvant treatment, *n* = 3; adjuvant treatment, *n* = 3

^e^ In the lymph node-positive group (N+), 67 were N1 and 35 were N2. Median number of lymph nodes in the N0 group was 14 (8–53)

^f^ Neoadjuvant treatment before liver resection (FOLFOX, *n* = 69; FOLFIRI, *n* = 3; FOLFIRI-bevacizumab, *n* = 7; folinic acid-fluorouracil, *n* = 2)

^g^ Adjuvant treatment after liver resection (FOLFOX, *n* = 74; FOLFIRI, *n* = 1; FOLFIRI-bevacizumab, *n* = 12; folinic acid-fluorouracil, *n* = 2)

### Statistical Methods

Data were analyzed using IBM SPSS version 20 (IBM Corporation, Armonk, NY, USA) and STATA 11.0 (StataCorp LP, College Station, TX, USA). Differences in continuous variables were tested using an independent sample *t* test and the Pearson Chi square test for contingency tables. The Fisher’s exact test was used to compare ratios. An explanatory strategy was used to investigate the relationship between N status and survival. All other variables were only of interest as possible confounders or effect modifiers of this association. A Mantel–Haenszel stratification analysis using the patient years (time) model was performed to quantify confounders and to pinpoint effect modifiers, using the Breslow–Day test of heterogeneity.

Adjustment for multiple confounders was carried out using the Cox proportional hazard regression model with a manual backward elimination procedure. Kaplan–Meier survival curves were used to determine differences in survival between N0/N+ , and outcomes were recorded as recurrence-free survival (RFS) and overall survival (OS). OS/RFS was defined as the number of days from resection of CLM to death/radiological recurrence. The association between N status and survival was quantified by hazard ratio (HR) with its 95% confidence interval (CI). Statistical significance was set at *p* < 0.050.

## Results

### Patients

Table 1 summarizes the characteristics of the 140 CLM patients, with 27.1% N0 (*n* = 38) and 72.9% N+ (*n* = 102) tumors. A T2 primary tumor was significantly more common in the N0 group (29.0% vs. 2.9% in the N+ group, *p* = 0.001) and primary colon cancer had a higher frequency of N0 status than primary rectal cancer (*p* = 0.007). Patients with RFS of more than 6 months between the primary tumor resection and liver metastases were more prone to have N0 primary cancer (60.5%), and, correspondingly, patients with <6 months RFS were more prone to have N+ disease (68.7%, *p* = 0.002). Fifteen patients in the N0 group (15/38) and 66 patients in the N+ group (66/102) received neoadjuvant treatment before liver resection. Patients were followed until the date of death or end of/last follow-up. The median follow-up time was 24 (1–61) months.

### CTC and DTC Detection

CTCs were detected in 12.1% of all patients; 5.3% in the N0 group and 14.7% in the N+ group (*p* = 0.156). Of the 17 CTC-positive patients, the number of CTCs detected were one in four (four patients), two CTCs (four patients), three CTCs (three patients), four CTCs (four patients) and five CTCs (two patients).

Interestingly, 80.2% (69/86) of the CTC-negative patients received neoadjuvant chemotherapy to the primary tumor compared with 60% (9/15) in the CTC-positive patients, supporting the sterilization effect of chemotherapy. However, in CTC-negative patients, 62.4% (53/85) received neoadjuvant treatment before liver resection compared with 92.9% (13/14) of the CTC-positive patients. To date, in this study a sterilization effect of chemotherapy on CTCs cannot be confirmed (data not shown).

DTCs were present in 8.6% of all patients; 10.5% in the N0 group and 7.8% in the N+ group (*p* = 0.734). DTC presence had no impact on either RFS or OS.

### Recurrence-Free Survival

**Fig. 2:**
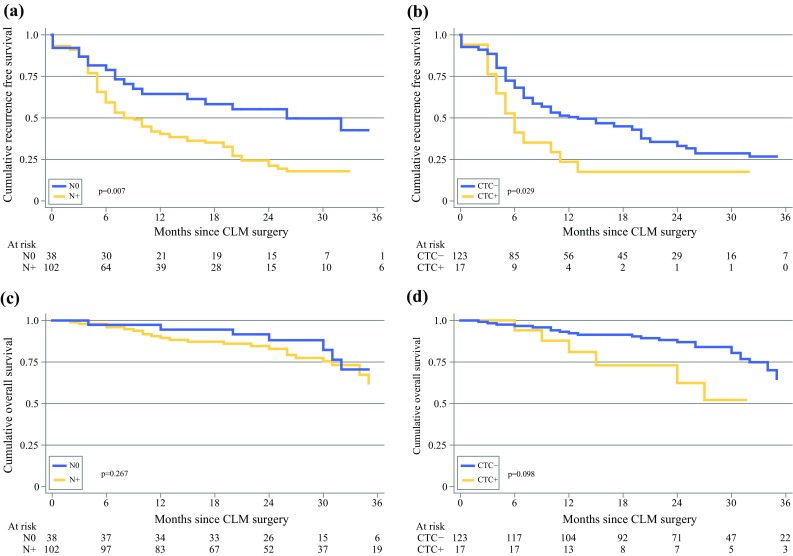
(**a**) Recurrence-free survival according to lymph node status (N+/N0) in 140 patients with resectable CLM (*p* < 0.01). (**b**) Recurrence-free survival according to CTC status in peripheral blood detected at liver surgery in 140 patients with resectable CLM (*p* = 0.029). (**c**) Overall survival analysing lymph node status (N+/N0) in 140 patients with resectable CLM (*p* = 0.267). (**d**) Overall survival according to CTC status in the peripheral blood at liver surgery in 140 patients with resectable CLM (*p* = 0.098). *CLM* colorectal liver metastases, *CTC* circulating tumor cells

Kaplan–Meier curves show a significant difference in RFS comparing N0 patients with N+ patients (26 vs. 9 months, *p* = 0.007) [Fig. 2a]. There was also a significant difference in RFS between CTC-positive and CTC-negative patients (6 vs. 13 months, *p* = 0.029) [Fig. 2b].

The crude HR for N status of the primary tumor and RFS after resection of the CLM was 1.95 (95% CI 1.17–3.23, *p* = 0.010). Potential confounders of the association between N status and RFS were identified, and the results of the stratified analysis are shown in electronic supplementary Table 1.

In the stratified analysis, the timing and number of liver metastases, in addition to neoadjuvant chemotherapy, were identified as possible confounders (confounding effect >8%). By multivariate Cox regression analysis, the number and timing of liver metastasis were identified as the strongest confounders (Table 2).

**Table 2:** Association between N+/N0 and recurrence-free survival after adjusting for multi-confounders in a cohort of 140 patients with CLM (multivariate analysis)

	Level	HR	95% CI	Standard error	*p*-Value
N status	N+/N0	1.78	1.02–3.13	0.43	0.04
No. of liver metastases	>3/1–3	1.92	1.17–3.16	0.32	0.009
Synchronous liver metastasis	Yes/no	1.45	0.91–2.31	1.08	0.12

*CLM* colorectal liver metastases, *HR* hazard ratio, *CI* confidence interval

Controlling for multiple confounding, the association between N status and RFS remained statistically significant (adjusted HR [HR_adj_] 1.74, 95% CI 1.07–2.83, *p* = 0.03). Overall, an association was noted between N+/N0 and synchronous/metachronous liver metastases and RFS (*p* = 0.006; electronic supplementary Fig. 1).

### Overall Survival

**Fig. 3:**
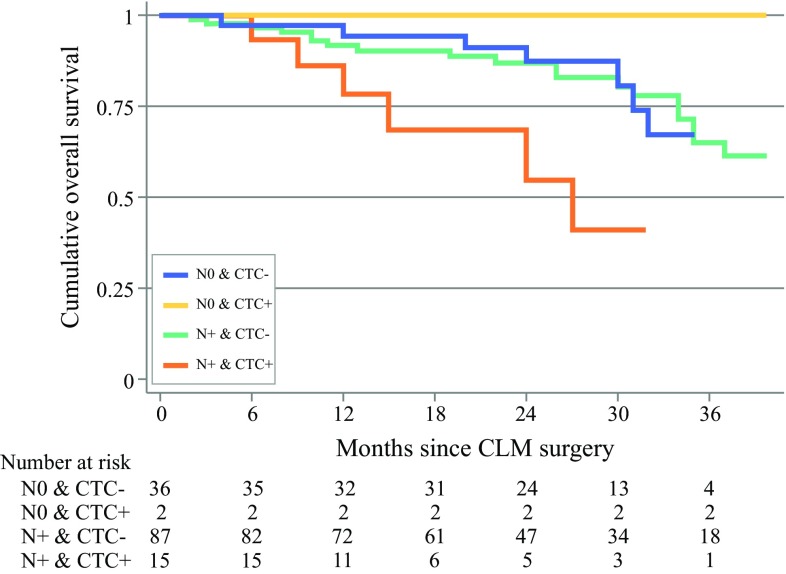
Overall survival analyzing the combination of lymph node status (N+/N0) and CTC status at liver surgery in 140 patients with resectable CLM. *CTC* circulating tumor cells, *CLM* colorectal liver metastases

Kaplan–Meier analyses showed no significant difference in OS between N0 and N+ patients (Fig. 2c). Analysing CTC status, no significant difference in OS between positive and negative patients was detected (53 vs. 52 months, *p* = 0.098) [Fig. 2d]. However, studying survival and CTC status in N+ patients, CTC-positive N+ patients had impaired survival compared with CTC-negative N+ patients (median survival 27 vs. 52 months, *p* = 0.024) [Fig. 3].

The crude HR for the association between N status and OS was 1.59 (95% CI 0.69–3.64, *p* = 0.274). Potential confounders were identified, and the results of the stratification analyses are shown in electronic supplementary Table 2.

In multivariate Cox regression analysis, CTC positivity and number of liver metastases were identified as the strongest confounders (Table 3). When controlling for multiple confounding, the association between N status and OS was not statistically significant (HR_adj_ 1.43, 95% CI 0.62–3.32, *p* = 0.065).

**Table 3:** Association between N+/N0 and overall survival after adjusting for multi-confounders in a cohort of 140 patients with CLM (multivariate analysis)

	Level	HR	95% CI	Standard error	*p*-Value
N status	N+/N0	1.43	0.61–3.32	0.55	0.40
No. of liver metastasis	>3/1–3	2.07	1.00–4.28	0.54	0.05
CTC-positive	Yes/No	2.31	1.00–4.28	0.66	0.05

*CLM* colorectal liver metastases, *HR* hazard ratio, *CI* confidence interval, *CTC* circulating tumor cell

Mortality in CTC-positive patients was 2.3-fold higher than in CTC-negative patients when adjusting for primary nodal status and number of liver metastases (Table 3).

## Discussion

In this study of resectable CLM patients, 27% of patients were N0 when examining their primary surgical specimen LNs. The frequency of CTC positivity at the time of CLM surgery was 12.1%, and was considerably higher in the N+ group compared with N0 patients. LN+ CTC-positive was associated with significant inferior RFS and OS. In the N0 group, only 5.3% of patients were CTC-positive; however, nodal status at primary surgery was not significantly associated with a difference in OS (Fig. 2). The presence of one or more CTCs at the time of CLM surgery was one of the strongest covariates for RFS and OS. This is in agreement with previous reports, suggesting CTCs are equal to, or even superior to, the conventional staging tools in estimating prognosis.^15^^,^ 24 The combination of CTC and nodal status was superior to either variable individually.^25^

The presence of LN metastases is a well-recognized prognostic factor^26^^–^ 28 but the optimal number of LNs required for adequate staging is uncertain. Evaluation of at least 12 nodes is widely cited in clinical guidelines.^29^^–^ 31 LN harvest is affected by several factors, such as microsatellite instability and tumor location.^32^ Rhabari et al. showed that molecular detection of tumor cells in regional LNs was associated with an increased risk of disease recurrence and poor survival in patients with apparently node-negative CRC. This emphasizes the importance of adequate molecular examination of the retrieved nodes for correct staging.^33^

In spite of general improvement in diagnostics, prognostic tools, and surgical and pathological evaluation, 25–50% of CRC patients experience recurrence. On the other hand, 50% of node-positive CRC patients seem to be cured by surgery alone. Adjuvant treatment has been proven to increase survival in non-metastatic CRC,^34^^,^ 35 but the survival benefit of chemotherapy treatment varies.^36^ Selection criteria for chemotherapy and targeted treatment are warranted, and biological markers may contribute to this.^37^

CTCs are components of the metastatic cascade, and CTC presence has proven to be a strong predictor of survival in patients with early and metastatic epithelial cancer, including CRC.^38^^–^ 41 The impact of CTCs on survival in CRC has been reported in a meta-analysis by Rahbari et al. but the study has been criticized for methodological heterogeneity.^40^ A recent meta-analysis by Huang et al. confirms the prognostic significance of CTCs detected using the CellSearch system in CRC.^24^ Huang et al. conclude that uncertainties still remain regarding the optimal sampling time for CTC analyses to provide the most accurate prognostic information. Serial sampling seems to represent an important tool for monitoring treatment.^42^^,^ 43

The cut-off value for CTC positivity differs between studies. The impact of the number of CTCs on survival has been published by our group.^17^ The low cut-off value in this study (one or more CTCs per 7.5 ml of blood) was chosen because the study included patients being treated with chemotherapy, knowing the negative impact of such treatment on CTC levels,^44^ as also reported in the present study. Thus, the cut-off value of being CTC-positive in metastatic CRC patients is not yet settled.^45^

In this study, 15 of the N0 patients had synchronous liver metastases and all received neoadjuvant chemotherapy. These patients had a significantly better RFS than N+ patients with synchronous liver metastases (electronic supplementary Fig. 1). The high frequency of metastases in the N0 patients supports the view that cancer cell contamination occurs at different stages in cancer development. In N0 patients with synchronous liver metastases, the tumor cells have bypassed the LNs, and one could hypothesize that the disease may be more chemoresponsive when limited to the liver.^46^^,^ 47 These patients may be prime candidates for neoadjuvant treatment.

Supported by the results in our study, a possible approach to improve prognostic accuracy could be to combine CTC detection and N staging, as also suggested by Allen-Mersh et al.^25^ and Van Dalum et al.^48^ This might improve selection to adjuvant chemotherapy in both LN-negative and LN-positive CRC patients. Serial CTC monitoring during follow-up may further improve surveillance of metastatic CRC patients.^48^ Our recent results indicate the potential impact of detection of CTCs for prognosis and recurrence through serial monitoring.^47^ In addition, further molecular characterization of CTCs to identify therapeutic targets opens the possibility of tailoring CRC treatment individually.

The presence of DTCs at primary surgery is reported to be a prognostic biomarker of impaired survival in patients with CRC in long-term follow-up.^18^ This may be explained by DTCs representing dormant tumor cells with the ability to later escape dormancy, proliferate, and cause relapse in apparently curatively resected cancer patients, suggesting a role for DTCs as a biomarker in CRC.^49^ In the present study, the presence of DTCs in CLM surgery did not seem to have an impact on survival, but the follow-up time was limited.

This study has several limitations. The short median follow-up is partly because 40.8% of the study population had died within 4 years of follow-up. More extensive follow-up would further elucidate the clinical outcome for these patients. Sampling of CTC and DTC was performed at the time of liver surgery. Thus, we cannot exclude the possibility that any chemotherapy given before sampling (adjuvant for the primary or neoadjuvant for the liver metastases) may have affected the CTC and DTC status. This could explain a relative low rate of positivity. Only a small number of N0 patients (*n* = 38) were analyzed, and the frequency of CTC positivity among these patients was low (5.3%). N0 CTC-positive patients appear to be a high-risk group but further studies are needed to confirm this.

Due to non-standardized pathology reports at the time of this study, more than 30% did not report vascular invasion. Unfortunately, the presence of vascular invasion could not be analyzed in the present study. Molecular analyses for characterization of CTCs as microsatellite instability and KRAS/BRAF mutation may also provide clinically relevant information for prognosis and treatment options in these patients, and should be further tested in clinical studies.

## Conclusion

This study shows the presence of CTCs is associated with impaired survival. CTC status seems to provide additive prognostic information to LN status in patients with CLM.

## Electronic supplementary material

Below is the link to the electronic supplementary material.

Supplementary material 1 (TIFF 2215 kb). Supplementary Figure 1a Recurrence free survival analysing the combination of lymph node status (N+/N0) and synchronous versus metachronous liver metastases in 140 patients with resectable colorectal liver metastases, p = 0.006. b Recurrence free survival analysing the combination of lymph node status (N+/N0) and synchronous versus metachronous liver metastases adjusted for neoadjuvant treatment in 140 patients with resectable colorectal liver metastases, p = 0.087

Supplementary material 2 (DOC 78 kb)

Supplementary material 3 (DOC 74 kb)
