# Supplementary figures and images for: Survival Impact of Primary Tumor Lymph Node Status and Circulating Tumor Cells in Patients with Colorectal Liver Metastases
Source: Ann Surg Oncol. 2017 Mar 3;24(8):2113–21. doi: 10.1245/s10434-017-5818-2 (PMC5491630; doi:10.1245/s10434-017-5818-2)

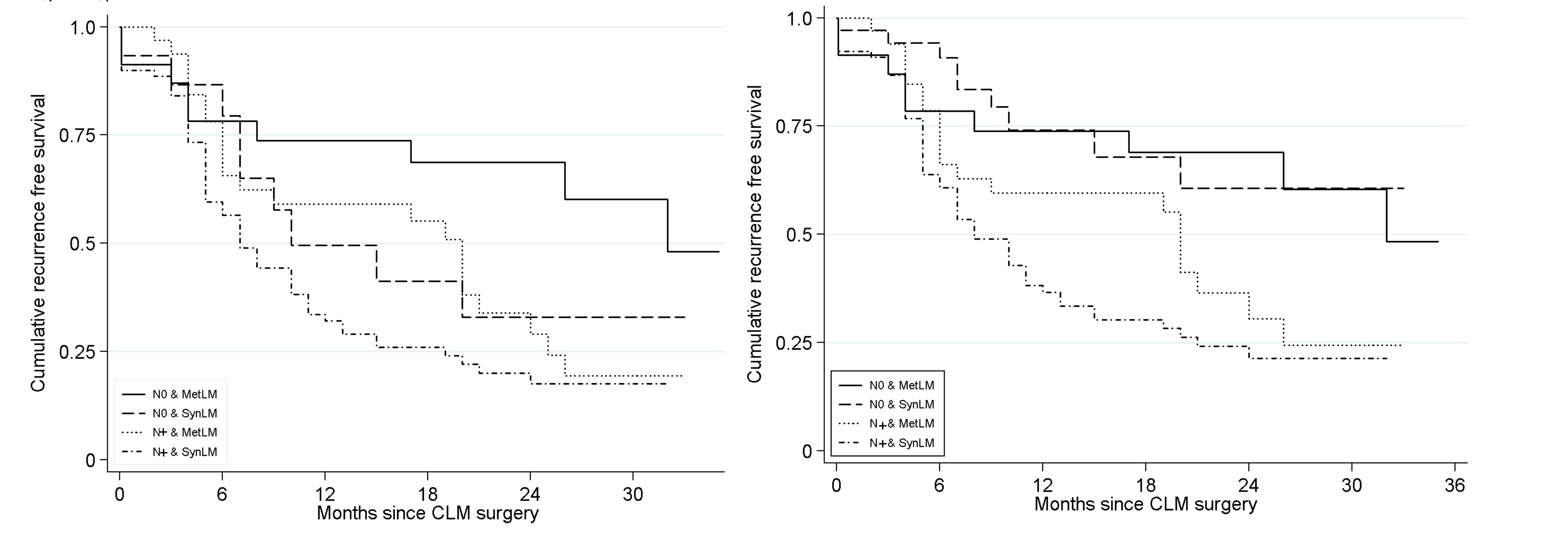

Supplement: Supplementary file 1 — Supplementary material 1 (TIFF 2215 kb). Supplementary Figure 1a Recurrence free survival analysing the combination of lymph node status (N+/N0) and synchronous versus metachronous liver metastases in 140 patients with resectable colorectal liver metastases, p = 0.006. b Recurrence free survival analysing the combination of lymph node status (N+/N0) and synchronous versus metachronous liver metastases adjusted for neoadjuvant treatment in 140 patients with resectable colorectal liver metastases, p = 0.087 [file 10434_2017_5818_MOESM1_ESM.tiff]
