# Supplementary material for: Survival Impact of Primary Tumor Lymph Node Status and Circulating Tumor Cells in Patients with Colorectal Liver Metastases
Source: Ann Surg Oncol. 2017 Mar 3;24(8):2113–21. doi: 10.1245/s10434-017-5818-2 (PMC5491630; doi:10.1245/s10434-017-5818-2)
Supplement: Supplementary file 2 — Supplementary material 2 (DOC 78 kb) [file 10434_2017_5818_MOESM2_ESM.doc]

| **Supplementary Table 1: Stratified analysis of the association between N status and recurrence free survival (RFS)** | | | | | | | |
| --- | --- | --- | --- | --- | --- | --- | --- |
|  | **N+** | | **N0** | |  |  |  |
|  | Number of recurrences | Person – months | Number of recurrences | Person – months | IRR  (95% CI) | IRR_MH_  (95% CI) | Heterogeneity test (P-value) |
| Crude | 75 | 1263 | 19 | 646 | 2.02 (1.21 – 3.54) |  |  |
| **Age (years)** |  |  |  |  |  |  |  |
| >65 | 40 | 729 | 10 | 330 | 2.30 (1.09 – 5.45) | 2.03 (1.23 – 3.36) | 0.64 |
| ≤65 | 35 | 533 | 9 | 316 | 1.81 (0.89 – 4.06) |  |  |
| Sex |  |  |  |  |  |  |  |
| Male | 41 | 661 | 14 | 298 | 1.31 (0.70 – 2.62) | 1.96 (1.19 – 3.23) | 0.05 |
| Female | 34 | 601 | 5 | 348 | 3.93 (1.53 – 12.89) |  |  |
| Liver metastasis |  |  |  |  |  |  |  |
| Synchronous | 53 | 765 | 10 | 209 | 1.44 (0.73 – 3.19) | 1.71 (1.03 – 2.84) | 0.45 |
| Metachronous | 22 | 498 | 9 | 437 | 2.14 (0.95 – 5.29) |  |  |
| **Neoadjuant Chemotherapy** |  |  |  |  |  |  |  |
| Yes | 50 | 753 | 10 | 207 | 1.37 (0.69 – 3.04) | 1.72 (1.03 – 2.85) | 0.32 |
| No | 22 | 467 | 9 | 439 | 2.29 (1.01 – 5.66) |  |  |
| T status |  |  |  |  |  |  |  |
| T2 | 71 | 1167 | 14 | 477 | 2.07 (1.15 – 3.98) | 1.91 (1.12 – 3.28) | 0.35 |
| T3-T4 | 2 | 72 | 5 | 163 | 0.91 (0.09 – 5.53) |  |  |
| CTC |  |  |  |  |  |  |  |
| Positive | 12 | 139 | 2 | 9 | 0.38 (0.08 – 3.57) | 1.84 (1.12 – 3.03) | 0.03 |
| Negative | 63 | 1124 | 17 | 637 | 2.10 (1.21 – 3.83) |  |  |
| DTC |  |  |  |  |  |  |  |
| Positive | 4 | 91 | 2 | 35 | 0.76 (0.11 – 8.49) | 2-06 (1.21 – 3.50) | 0.24 |
| Negative | 65 | 1070 | 15 | 557 | 2.25 (1.27 – 4.25) |  |  |
| ECOG |  |  |  |  |  |  |  |
| ≥1 | 6 | 105 | 2 | 139 | 3.97 (0.71 – 40.23) | 1.92 (1.16 – 3.18) | 0.35 |
| 0 | 69 | 1158 | 17 | 507 | 1.77 81.03 – 3.22) |  |  |
| Primary tumour |  |  |  |  |  |  |  |
| Colon | 46 | 757 | 17 | 537 | 1.91 (1.07 – 3.56 ) | 2.09 (1.24 – 3.52) | 0.52 |
| Rectum | 29 | 505 | 2 | 110 | 3.15 (0.79 – 27.31) |  |  |
| CEA |  |  |  |  |  |  |  |
| <6 ng/ml | 32 | 610 | 8 | 341 | 2.23 (1.00 – 5.61) | 2.02 (1.17 – 3.50) | 0.72 |
| ≥6 ng/ml | 32 | 466 | 8 | 214 | 1.83 (0.82 – 4.61) |  |  |
| **Number of liver metastases** |  |  |  |  |  |  |  |
| 1-3 | 52 | 1026 | 16 | 616 | 1.95 (1.09 – 3.66) | 1.74 (1.05 – 2.89) | 0.304 |
| >3 | 23 | 236 | 3 | 30 | 0.97 (0.29 – 5.06) |  |  |
| Primary adjuvant therapy |  |  |  |  |  |  |  |
| Yes | 55 | 992 | 1 | 32 | 1.77 (0.30 – 71.29) | 2.77 (1.51 – 5.10) | 0.621 |
| No | 20 | 229 | 18 | 614 | 2.97 (1.49 – 5.97) |  |  |
| Poor histological differentiation^a^ |  |  |  |  |  |  |  |
| Yes | 8 | 116 | 0 | 33 | 4.55 (0.26 – 79.24) | 1.90 (1.15 – 3.14) | 0.536 |
| No | 67 | 1147 | 19 | 592 | 1.82 (3.21 – 12.53) |  |  |

^a^The zero cell has been replaced by 0.5 to be able to calculate the stratum – specific IRR, the adjusted Mantel – Haenszel IRR and the Breslow and Day test of heterogeneity.

Confounding effect is quantified using the formula $\frac{IRR_{MH}-IRR_{crude}}{IRR_{crude}}$
