# Supplementary material for: Survival Impact of Primary Tumor Lymph Node Status and Circulating Tumor Cells in Patients with Colorectal Liver Metastases
Source: Ann Surg Oncol. 2017 Mar 3;24(8):2113–21. doi: 10.1245/s10434-017-5818-2 (PMC5491630; doi:10.1245/s10434-017-5818-2)
Supplement: Supplementary file 3 — Supplementary material 3 (DOC 74 kb) [file 10434_2017_5818_MOESM3_ESM.doc]

| **Supplementary Table 2: Stratified analysis of the association between N status and overall survival (OS)** | | | | | | | |
| --- | --- | --- | --- | --- | --- | --- | --- |
|  | **N+** | | **N0** | |  |  |  |
|  | Number of deaths | Person–months | Number of deaths | Person–months | IRR  (95% CI) | IRR_MH_  (95% CI) | Heterogeneity test (P-value) |
| Crude | 28 | 2527 | 7 | 1038 | 1.64 (0.70 – 4.46) |  |  |
| **Age (years)** |  |  |  |  |  |  |  |
| >65 | 14 | 1477 | 3 | 551 | 1.74 (0.49 - 9.44) | 1.67 (0.73 – 3.84) | 0.94 |
| ≤65 | 14 | 1050 | 4 | 487 | 1.62 (0.51 – 6.77) |  |  |
| Sex |  |  |  |  |  |  |  |
| Male | 14 | 1212 | 5 | 589 | 1.36 (0.46 – 4.82 ) | 1.68 (0.73 – 3.87) | 0.54 |
| Female | 14 | 1315 | 2 | 449 | 2.39 (0.55 – 21.66) |  |  |
| Liver metastasis |  |  |  |  |  |  |  |
| Synchronous | 18 | 1691 | 3 | 423 | 1.50 (0.43 – 7.95) | 1.66 (0.72 – 3.86) | 0.83 |
| Metachronous | 10 | 836 | 4 | 615 | 1.83 (o.53 – 8.03) |  |  |
| **Neoadjuant Chemotherapy** |  |  |  |  |  |  |  |
| Yes | 18 | 1511 | 4 | 406 | 1.21 (0.39 – 4.91) | 1.53 (0.67 – 3.51) | 0.52 |
| No | 9 | 893 | 3 | 632 | 2.12 (0.53 – 12.20) |  |  |
| T status |  |  |  |  |  |  |  |
| T3-T4 | 25 | 2360 | 4 | 743 | 1.96 (0.68 – 7.77) | 1.73 (0.67 – 4.47) | 0.53 |
| T2 | 1 | 110 | 3 | 289 | 0.87 (0.01 – 10.9) |  |  |
| CTC |  |  |  |  |  |  |  |
| Positive | 7 | 292 | 0 | 92 | 4.11 (0.25 – 77.7) | 1.34 (0.85 – 2.00) | 0.40 |
| Negative | 21 | 2235 | 7 | 946 | 1.27 (0.52 – 3.54) |  |  |
| DTC |  |  |  |  |  |  |  |
| Positive | 3 | 155 | 1 | 59 | 1.14 (0.09 – 59.9) | 1.43 (0.62 – 3.31) | 0.83 |
| Negative | 23 | 2184 | 6 | 848 | 1.48 (0.59 – 4.46) |  |  |
| ECOG |  |  |  |  |  |  |  |
| ≥1 | 2 | 171 | 1 | 163 | 1.90 (0.09 – 112.5) | 1.64 (0.71 – 3.77) | 0.89 |
| 0 | 26 | 2356 | 6 | 875 | 1.60 (0.64 – 4.78) |  |  |
| Primary tumour |  |  |  |  |  |  |  |
| Colon | 19 | 1535 | 6 | 882 | 1.81 (0.69 – 5.56) | 1.74 (0.75 – 4.03) | 0.83 |
| Rectum | 9 | 992 | 1 | 156 | 1.41 (0.19 – 62.02) |  |  |
| CEA |  |  |  |  |  |  |  |
| <6 ng/ml | 8 | 1240 | 3 | 543 | 1.16 (0.28 – 6.83) | 1.63 (0.67 – 4.02) | 0.53 |
| >6 ng/ml | 16 | 944 | 3 | 371 | 2.09 (0.60 – 11.2) |  |  |
| **Number of liver metastases** |  |  |  |  |  |  |  |
| 1-3 | 17 | 1874 | 6 | 936 | 1.41 (0.53 – 4.38) | 1.46 (0.63 – 3.43) | 0.87 |
| >3 | 11 | 653 | 1 | 102 | 1.71 (0.25 – 73.9) |  |  |
| Primary adjuvant therapy |  |  |  |  |  |  |  |
| Yes | 20 | 1913 | 0 | 47 | 0.98 (0.06 – 16.3) | 1.82 (0.70 – 4.73) | 0.65 |
| No | 8 | 573 | 7 | 991 | 1.98 (0.63 – 6.40) |  |  |
| Poor histological differentiation^a^ |  |  |  |  |  |  |  |
| Yes | 5 | 240 | 0 | 33 | 1.38 (0.08 – 25.2) | 1.41 (0.63 – 3.18) | 0.99 |
| No | 23 | 2287 | 7 | 984 | 1.41 (0.59 – 3.90) |  |  |

^a^The zero cell has been replaced by 0.5 to be able to calculate the stratum–specific IRR, the adjusted Mantel–Haenszel IRR and the Breslow-Day test of heterogeneity.

Confounding effect is quantified using the formula $\frac{IRR_{MH}-IRR_{crude}}{IRR_{crude}}$
